# Reversal of left-sided colostomy utilizing single-port laparoscopy a multicenter European audit and overview of the literature

**DOI:** 10.1007/s00464-021-08657-x

**Published:** 2021-07-26

**Authors:** Y. T. van Loon, S. H. E. M. Clermonts, E. H. J. Belgers, H. Kurihara, A. Spinelli, H. M. Joshi, K. J. Gorissen, D. D. E. Zimmerman

**Affiliations:** 1 Department of Surgery, Elisabeth-TweeSteden Hospital, Hilvarenbeekseweg 60, 5022GC Tilburg, The Netherlands; 2 Department of Surgery, Maastricht University Medical Center, Maastricht, The Netherlands; 3 Department of Surgery, Zuyderland Hospital, Heerlen and Sittard, The Netherlands; 4 Department of Colorectal Surgery, Humanitas University, Milan, Italy; 5 Department of Emergency Surgery and Trauma Unit, Humanitas Clinical and Research Center, IRCCS, Rozzano, Milan, Italy; 6 Department of Colon and Rectal Surgery, Humanitas Clinical and Research Center IRCCS, Rozzano, Milan, Italy; 7 Department of Biomedical Sciences, Humanitas University, Pieve Emanuele, Rozzano, Milan, Italy; 8 Department of Colorectal Surgery, Addenbrooke’s Hospital, Cambridge University Hospitals NHS Foundation Trust, Cambridge, UK; 9 Department of Emergency and Colorectal Surgery, Oxford University Hospitals NHS Foundation Trust, Oxford, UK

**Keywords:** Hartmann’s procedure, Single-port laparoscopy, Stoma reversal, Hartmann reversal, Single-incision

## Abstract

**Background:**

Stoma reversal surgery can result in considerable morbidity and even mortality. Feasibility of utilizing single-port laparoscopy through the stoma fenestration have been shown before. Aim of the present observational study is to evaluate multicenter experiences of single-port reversal of left-sided colostomy (SPRLC) throughout Europe and to provide an overview of available literature on this topic.

**Methods:**

All patients undergoing SPRLC in four different teaching hospitals throughout Europe are included. Primary outcome was 30-day postoperative complication rate. Secondary outcomes were postoperative length of stay (LOS), single-port success rate and conversion rates. Appraisal of the available literature in PubMed was performed.

**Results:**

Of 156 SPRLC procedures, 98.7% of them were technically successful and 71.8% were without postoperative complications. No postoperative mortality was encountered. Superficial site infection occurred in 14.7%, anastomotic leakage in 3.9% and major complications in 8.3%. Median LOS was 4.0 days (1–69), single-port success rate was 64.7%, 12.8% and 21.2% (33/154) were converted to an open and multiport laparoscopic procedure, respectively. Literature shows equally favorable results in 131 patients divided over 5 cohorts with morbidity ranging from 0 to 30.4% and mortality from 0 to 2.2% and median LOS of 4–8 days.

**Conclusion:**

This study confirms the safety, feasibility and favorable results of the use of single-port approach in the reversal of left-sided colostomy in different centers in Europe with laparoscopic experienced colorectal surgeons. The available literature on this topic support and show equally favorable results using single-port laparoscopy for left-sided colostomy reversal surgery.

**Supplementary Information:**

The online version contains supplementary material available at 10.1007/s00464-021-08657-x.

Stomas are not only used in emergency colorectal surgery for benign disorders such as diverticulitis after a Hartmann’s Procedure (HP) [1–3]; they are also widely accepted and propagated in colorectal cancer surgery. Up to 35% of the Dutch elderly patients still receive an ostomy after colorectal cancer surgery and patients undergoing emergency colorectal surgery because of left-sided colon malignancy still suffer from significantly higher odds of a colostomy [4–6]

Pursuing stoma reversal surgery is not without risks; anastomotic leakage rates range from 4 to 16%, perioperative mortality and morbidity rates are reported as high as 14% and 40%, respectively [7–10]. These are the main reasons why many surgeons are reluctant to perform stoma reversal surgery; in up to 40% of the patients, stoma reversal will never be performed [8–10].

In 1993 laparoscopic reversal of HP was introduced in an effort to reduce morbidity and mortality [11]. It was shown to have considerable advantages over classic open reversal of HP [11, 12]. Further evolution of minimal invasive abdominal surgery introduced the use of single-port laparoscopy, this approach has also been used for stoma reversal surgery, first described by Smith and Bettinger [13]. The necessity of difficult trocar placement and laborious midline adhesiolysis are obvious advantages of the single-port approach over the conventional laparoscopic approach [14, 15]. Previous studies show that single-port reversal of a left-sided colostomy (SPRLC) is feasible, safe and also results in significant shorter length of stay and reduction of postoperative complications as superficial site infections compared to classic open stoma reversal surgery [3, 15]. Adoption of this approach has led to an increasing body of literature on SPRLC since its introduction in 2011.

Aim of the present study was to evaluate the results of single-port reversal of left-sided colostomy (SPRLC) in a multicenter setting across different countries in Europe and to give a comprehensive overview of the literature on the use of the single-port (SP) approach in the reversal of a left-sided colostomy. We postulate that SPRLC is feasible and safe with a shorter hospital stay and less postoperative morbidity compared to an open approach.

## Materials and methods

This is a retrospective observational study, all patients undergoing SPRLC in four different teaching hospitals throughout Europe were included in present analysis. The four different hospitals are Churchill Hospital in the United Kingdom (CH), Humanitas Research Hospital (HRH) in Italy, Elisabeth-TweeSteden Hospital (ETH) and Zuyderland Medical Center (ZMC) both in the Netherlands. Approval of the institutional review board or ethics committee was not required because of the retrospective and observational character of this study. This report was prepared in concordance with the STROBE guidelines [16].

Patient characteristics (sex, age, length and weight), index surgery characteristics (reason for surgery and postoperative complications), surgical details (time interval between index surgery and SPRLC, duration of SPRLC, conversion to multiport laparoscopy or laparotomy, colostomy site closure methods) and postoperative outcomes (length of stay, complications, readmissions) were collected in electronic case report forms by local investigators using the electronic patient records. All procedures were performed or supervised by experienced colorectal surgeons or consultants with extensive skills in laparoscopy and minimally invasive surgery. Patients undergoing stoma reversal of a loop or right-sided colostomy or ileostomy or via open procedure were excluded. This study included cases previously published by authors from our current collaborative group from Clermonts and van Loon (ETH) and Joshi (CH) [3, 15, 17]. In CH and ETH all consecutive patients eligible for HP reversal were included, without additional patient selection or exclusion in the enrollment period from 2010–2019 and 2012–2020, respectively. Patients included from HRH (2008–2019) and ZMC (2015–2018) were selected by their operating surgeon by the surgeon’s preference, in these hospitals all procedures were performed by one (supervising) surgeon. In HRH patients included in this study comprised almost half of all the patients who underwent stoma reversal surgery. Single-port laparoscopy is the preferred approach of choice in CH and ETH when performing stoma reversal surgery for left-sided colostomies. SPRLC became the preferred approach of choice towards the second half of the study period in HRH. The preferred approach for stoma reversal surgery in ZMC is surgeon dependent.

### Surgical technique

All patients were placed in modified lithotomy position and given metronidazole 500 mg and cefuroxime 1500 mg intravenously. The operative procedure of the SPRLC has been described in detail previously [3, 13–15, 17–19]. In short, the colostomy was mobilized down to the fascia and the anvil for a circular stapler was placed in the descending colon before returning it to the abdominal cavity through the original colostomy site. Pneumoperitoneum was established after placement of a single-port device or surgical glove-port. Where necessary, the splenic flexure or transverse colon was mobilized and adhesiolysis was performed under direct vision. Continuity was restored after adhesiolysis and proper visualization of the rectal stump with the use of a circular stapler. An air leak test was performed before port removal. Fascia and skin at colostomy site were closed as deemed appropriate. See Table 1 for a detailed description of the materials and techniques used during SPRLC.

**Table 1:** Overview of materials used during single-port reversal of left-sided colostomy

Center, Country	Type of stapler	Type of single-port access	Closure of fascia	Closure of skin
Humanitas, Italy	EEA28™ EEA31™	GelPOINT™	Interrupted stitches, Vicryl	Sutures and staples
Churchill, United Kingdom	CDH29A	Surgical glove-port	Running suture, PDS	Skin glue, staples and sutures, Monocryl
Zuyderland, Netherlands	CDH29A	OCTO™Port, surgical glove-port	Running suture, PDS	Intracutaneous purse-string suture, Vicryl
Elisabeth-TweeSteden, Netherlands	CDH29A, EEA™^,a^	GelPOINT™	Running suture, PDS	Intracutaneous, purse-string sutures, Monocryl

EEA™ circular stapler, Medtronic CDH29A, Ethicon J&J

GelPOINT™, Applied Medical

OCTO^TM^Port, Dalim SurgNet, Frankenman

Vicryl: polyglactin suture, Ethicon J&J; PDS: polydioxanone suture, Ethicon J&J; Monocryl: polyglactin suture, Ethicon J&J

^a^This hospital switched to the EEA™ stapler from 2017

All patients were treated within an established Enhanced Recovery After Surgery (ERAS) protocol [20]. Important components of the ERAS protocol applied similarly in all centers are antimicrobial prophylaxis with skin preparation, perioperative near-zero fluid balance, no use of pelvic, peritoneal or nasogastric drains or tubes, multimodal analgesia without use of NSAIDs, early postoperative mobilization and oral diet. Patients were discharged from the hospital when they were able to tolerate normal food, pass stool, were able to mobilize at a level that was similar to preoperative levels of mobilization and had adequate control of pain with use of oral analgesia. Minimum follow up period consisted of 30 days postoperatively.

### Outcomes

Primary outcome was 30-days postoperative complication defined as infections (surgical site, intra-abdominal abscess), urogenital complications (urinary tract infection, urine retention), ileus or gastroparesis, pulmonary complications (pneumonia, exacerbation) and blood-related complications (rectal blood loss, thrombosis or hematoma in wound or anastomosis) was classified using the Clavien–Dindo score. Clavien–Dindo grade 3 or higher were considered major complications in this analysis.

Secondary outcomes were postoperative length of stay (LOS), technical and single-port success rate, other surgical details of the procedure such as duration, conversion to multiport laparoscopy or open and overall success rate of SPRLC. Technical success rate is defined as successful stoma reversal with creation of an anastomosis. Single-port success rate is defined as successful stoma reversal solely using the single-port technique without placement of additional laparoscopic trocars or conversion to open surgery. Placement of additional trocars besides the OCTO™ Port, GelPOINT Path Access Platform or single-site glove-port is considered conversion to multiport laparoscopy.

### Statistical analysis

Descriptive statistics were expressed as median and range (minimum, maximum) for continuous variables. The Pearson *χ*^2^ test or the Fisher exact tests, if appropriate, were used for categorical variables. Mann–Whitney *U* and Kruskal–Wallis test was used for continuous variables. Statistical analysis was performed using the SPSS software package version 26 (SPSS, Chicago, IL). All *p* values < 0.05 were considered statistically significant.

### Appraisal of the literature

A literature search for relevant literature from 2011 (the introduction of single-port reversal of HP) on was performed using PubMed. Articles were screened using title and abstract. When multiple articles from a single study group with matching authors was found, only most recent was used in an effort to reduce duplication bias. Previous published articles on this topic from our current collaborative group of authors were excluded.

## Results

A total of 156 patients were included from four different surgical departments throughout Europe: 30 patients from CH, 13 patients from HRH in Italy, 9 patients from ZMC and 104 patients from ETH.

The majority of the patients are male (m:f = 99:57), ASA 2 or 3 (40.4% and 34.6%, respectively) with a median age of 61.0 years (range 17.7–92.6). Majority of the index surgeries were via conventional open approach (64.7%), most common indications for index surgery were diverticulitis (58%) or colorectal cancer (23.7%). The median time between the index surgery and SPRLC is approximately 9 months (284 days). An overview of patient specific characteristics at baseline can be found in Table 2.

**Table 2:** Patient characteristics at baseline per center

Center, country	Number	Sex M:F	Median age (range)	Median BMI (range)	ASA *n* (%)		Reason for stoma n (%)		Primary open approach *n* (%)
Humanitas, Italy	13	9:4	64.5 (21.2–92.6)	24.4 (18.2–34.7)	1	1 (7.7)	Diverticulitis	4 (30.8)	10 (76.9)
					2	9 (69.2)	Malignancy	4 (30.8)	
					3	3 (23.1)	Perforation or trauma	3 (23.1)	
					4	–	Inflammatory Bowel Disease	1 (1.8)	
							Clostridium	1 (1.8)	
Churchill, United Kingdom	30	16:14	60.0 (17.7–80.1)	26.0 (19.0–45.2)			Diverticulitis	17 (56.7)	25 (83.3)
					1	1 (3.3)	Malignancy	5 (16.7)	
					2	7 (23.3)	Perforation or trauma	3 (10.0)	
					3	18 (60.0)	Anastomotic leakage	2 (6.7)	
					4	4 (13.3)	Ischemia	1 (3.3)	
							Volvulus	1 (3.3)	
							Stoma retraction	1 (3.3)	
Zuyderland, The Netherlands	9	6:3	56.1 (37.5–67.2)	25.6 (21.1–33.6)	1	2 (22.2)	Diverticulitis	4 (44.4)	6 (66.7)
					2	4 (44.4)	Malignancy	2 (22.2)	
					3	3 (33.3)	Volvulus	1 (11.1)	
					4	–	Ischemia	1 (11.1)	
							Perforation or trauma	1 (11.1)	
Elisabeth-TweeSteden, The Netherlands	104	68: 36	61.0 (25.5–85.0)	26.4 (18.3–61.1)			Diverticulitis	66 (63.5)	60 (57.7)
					1	25 (24.0)	Malignancy	26 (25.0)	
					2	43 (41.3)	Perforation or trauma	6 (5.7)	
					3	30 (28.8)	Ischemia	2 (1.9)	
					4	6 (5.8)	Volvulus	2 (1.9)	
							Inflammatory Bowel Disease	1 (1.0)	
							Perianal abscesses	1 (1.0)	
Cumulative	156	99: 57	61.0 (17.7–92.6)	26.3 (18.2–61.1)			Diverticulitis	91 (58.3)	101 (64.7)
							Malignancy	37 (23.7)	
					1	29 (18.6)	Perforation or trauma	13 (8.3)	
					2	63 (40.4)	Ischemia	4 (2.6)	
					3	54 (34.6)	Volvulus	4 (2.6)	
					4	10 (6.4)	Inflammatory Bowel Disease	2 (1.3)	
							Anastomotic leakage	2 (1.3)	
							Other	3 (1.9)	

### Postoperative results

No 30-day postoperative mortality was encountered in the present series. Majority (112/156, 71.8%) of the patients encountered no 30-day postoperative complications whatsoever, 28.2% of the patients encountered at least one postoperative complication. Major complications were encountered in 8.3%, Clavien Dindo grade 3 occurred in 5.7% (*n* = 9) and grade 4 in 2.6% (*n* = 4) of the patients. Anastomotic leakage rate was 3.9% (*n* = 6). Five of the patients with anastomotic leakage underwent reintervention under general anesthesia, two of them had their anastomosis disconnected into colostomies. The anastomosis of the other three patients could be salvaged by additional sutures at the staple line in one patient, additional stapling of the leaking rectal stump in one patient and drainage of the abscess and deviating ileostomy in one patient. One patient with anastomotic leakage presented with an intra-abdominal abscess which didn’t require a re-intervention. Surgical site infection (SSI) was the most frequent complications and occurred in 14.7% (*n* = 23). Six of the 23 patients with SSI developed this after conversion to open surgery, additional four of the 23 patients developed this after reoperation, these patients all had SSI of the laparotomy wound. The other 13 patients suffered from SSI of the old stoma incision. Overall median LOS was 4.0 days (range 1–69 days).

Four patients needed ICU admission, two were after anastomotic leakages, one patient with COPD suffered from a severe postoperative pneumonia and one patient needed rhythm observation due to severe tachycardia as a result of intra-abdominal abscess. None of these complications were deemed specific to the technique that was used, but are to be considered inherent to restoration of intestinal continuity procedures. Detailed overview of overall postoperative complications and complications per hospital can be found in Table 3.

**Table 3:** 30-day postoperative outcome

30-day postoperative outcome	Humanitas Italy (*n* = 13)	Churchill United Kingdom (*n* = 30)	Zuyderland Netherlands (*n* = 9)	Elisabeth-TweeSteden Netherlands (*n* = 104)	Cumulative (*n* = 156)
Median length of stay, days (range)	6 (3–19)	5 (2–35)	3 (1–11)	4 (1–69)	4 (1–69)
Any postoperative complication, *n* (%)	4 (30.8)	4 (13.3)	1 (11.1)	35 (33.7)	44 (28.2)
Anastomotic leakage, *n* (%)	1 (7.7)	–	1 (11.1)	4 (3.8)	6 (3.9)^c^
Surgical site infection, *n* (%)	–	1 (3.3)	1 (11.1)	21 (20.2)	23 (14.7)
Intra-abdominal abscess, *n* (%)	1 (7.7)	2 (6.7)	1 (11.1)	4 (3.8)	8 (5.1)
Urogenital complication^a^, *n* (%)	–	1 (3.3)	–	2 (1.9)	3 (1.9)
Postoperative ileus, *n* (%)	1 (7.7)	–	–	5 (4.8)	6 (3.9)
Pulmonary complication, *n* (%)	–	1 (3.3)	–	2 (1.9)	3 (1.9)
Bleeding-related complication^b^, *n* (%)	1 (7.7)	2 (6.7)	–	2 (1.9)	5 (3.2)
Clavien–Dindo classification, *n* (%)					
I	1 (7.7)	–	–	23 (22.1)	24 (15.4)
II	1 (7.7)	2 (6.7)	–	4 (3.8)	7 (4.5)
III	2 (15.4)	2 (6.7)	1 (11.1)	4 (3.8)	9 (5.7)
IV	–	–	–	4 (3.8)	4 (2.6)
Mortality, *n* (%)	–	–	–	–	–

^a^Urogenital complication: urine retention, urinary tract infection

^b^Bleeding-related: rectal blood loss, haematoma in wound or anastomosis

^c^Calculated over the number of patients who had successful reversal of left-sided colostomy, see Table 4

### Operative technique

Of the 156 procedures, two procedures (1.3%) were not technically successful in restoring intestinal continuity, resulting in a surgical success rate of 98.7%. Of the remaining 154 procedures, deviating stoma was needed in 2.6% of the procedures. SP approach was technically successful in 64.7% (101/156) of the procedures. Overall median operating time was 128 min (range 44–332). Conversion to multiport laparoscopy and open surgery was needed in 21.2% (*n* = 33) and 12.8% (*n* = 20), respectively. Additional ports were mostly needed for (extensive) adhesiolysis, oversewing the anastomosis after positive air leak testing or mobilizing the splenic flexure. Conversion to open surgery was significantly higher in patients who had an open index surgery compared to those who had a laparoscopic approach, 85.0% (17/20) of the conversions occurred in patients with open index surgery (*p* = 0.03), albeit single-port and multiport laparoscopy was feasible in 80.2% (81/101) of the patients who underwent open index surgery. Overview of the overall surgical details and outcomes and per hospital can be found in Table 4. Overview of the encountered intra-operative complications and reasons for conversion in ETH can be found in Online Appendix 1.

**Table 4:** Surgical details and outcomes

Surgical outcome	Humanitas Italy (*n* = 13)	Churchill United Kingdom (*n* = 30)	Zuyderland Netherlands (*n* = 9)	Elisabeth-TweeSteden Netherlands (*n* = 104)	Cumulative (*n* = 156)
Surgical success rate, *n* (%)	13 (100)	30 (100)	9 (100)	102 (98.1)	154 (98.7)
Deviating stoma, *n* (%)	–	1 (3.3)	1 (11.1)	2 (2.0)	4 (2.6)^a^
Single-port success rate, *n* (%)	13 (100)	12 (40.0)	9 (100)	67 (65.7)	101 (64.7)
Conversion	–	18 (60.0)	–	35 (34.3)	53 (34.0)
Multiport laparoscopy	–	12 (40.0)	–	21	33 (21.2)
Open	–	6 (20.0)	–	14	20 (12.8)
Median operation time [range]	160 [75–322]	165 [75–310]	88 [68–232]	128 [40–332]	128 [40–332]

^a^Calculated over the number of patients who had successful reversal of left-sided colostomy

### Appraisal of the literature

**Fig. 1:**
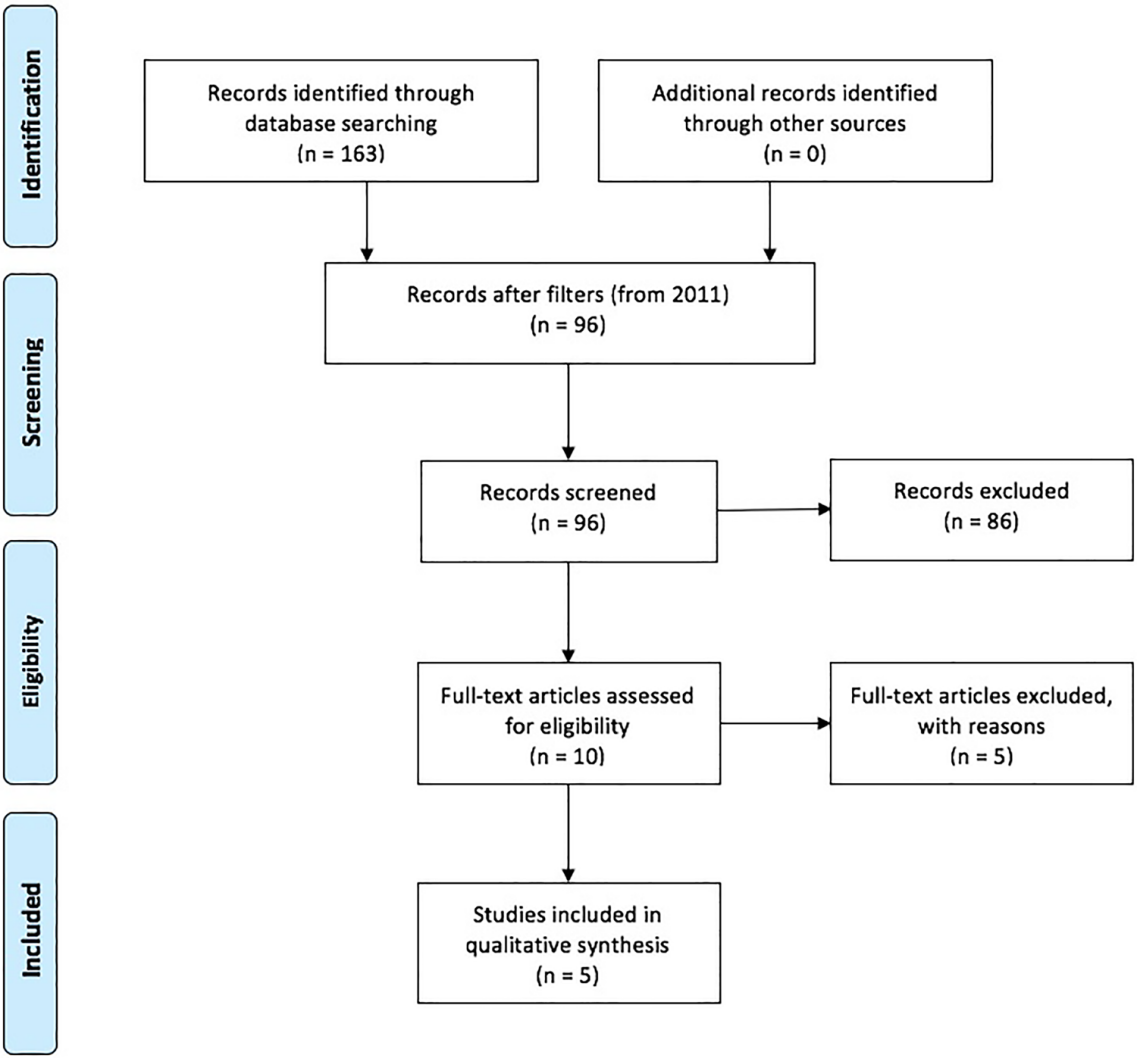
PRISMA flow diagram of included studies

The specific details of the literature search in PubMed can be found in Online Appendix 2. The flow diagram of inclusion of the studies can be found in Fig. 1. A total of 86 studies were excluded for solely discussing laparoscopy (*n* = 15), comparing results of laparoscopic reversal versus open reversal of left-sided colostomy (*n* = 11), treatment of diverticulitis (*n* = 25), video vignettes (*n* = 4), using other novel techniques of stoma reversal (*n* = 5), case reports on stoma problems (*n* = 3) and other articles unrelated to single-port stoma reversal surgery (*n* = 23).

Three previous published articles on this topic from our current collaborative group of authors were excluded [3, 15, 17], one article was excluded due to inclusion of their more recent manuscript [21], one meta-analysis on this topic was excluded since it had no additional new studies [22], leaving 5 included original articles [13, 14, 19, 23, 24].

This appraisal shows that since the introduction of the single-port approach in Hartmann’s reversal in 2011 by Smith and Bettinger [13], additional case series have been published on this topic. At this moment no randomized controlled trials between the different approaches (open, laparoscopic or single-port) were published. Literature shows that patient selection for SPRLC is mainly in patients after laparoscopic index surgery 53.4% overall, figures ranging from 34 to 72.7%. It also shows that SP approach is safe and feasible with high success rates, morbidity and mortality rates ranging from 12.5–30.4% and 1.8–2.2%, respectively. Major complication rate is also low, varying from 0–8.9% and median LOS between 4–8 days. Details and an overview can be found in Table 5.

**Table 5:** Summary of single-port reversal of left-sided colostomy in the current literature

Study	Country	Year of publication	Number of patients	Type of single-port access	Open index surgery	Conversion to laparoscopy	Conversion to open	Morbidity	Mortality	Median LOS (range)
Smith et al.	USA	2011	1	SSLAS	1 (100)	–	–	–	–	5^a^
Carus et al.	Germany	2011	8	SILS™ port	3 (37.5)	–	–	1 (12.5)	–	6.4 (4–8)
Choi et al.	Korea	2015	22	Surgical glove-port, OCTO™ Port	6 (27.3)	–	–	4 (18.2)	–	8 (4–31)
Thambi et al.	UK	2019	56^c^	GelPOINT™, SILS port, OCTO™ Port	37 (66.1)	1 (1.8)	5 (8.9)	17 (30.4)	1 (1.8)	4 (2–44)
D’Alessandro et al.	France	2020	44	GelPOINT™	14 (31.8)	6 (13.6)	–	7 (15.9)	1 (2.2)	4.8^b^
Cumulative			131		61 (46.6)	7 (5.3)	5 (3.8)	29 (22.1)	2 (1.5)	4–8

*LOS* length of stay

SSLAS (Single-Site laparoscopic access system), Ethicon SILS^TM^Port, Covidien GelPOINT™, Applied Medical OCTO^TM^Port, Dalim SurgNet, Frankenman

^a^No range

^b^No range could be found in the results or discussion section of this article

^c^3 patients had a single incision Hartmann’s procedure

## Discussion

The merits of the single-port reversal of left-sided colostomy compared to the open approach have been shown before [3, 11–15, 17, 19–21]. The present series is, to our knowledge, the only and largest European multicenter cohort to date. This study shows that SPRLC is an attractive technique and with favorable postoperative outcomes across different hospitals in Europe. Acceptable rates of postoperative morbidity (28%) and low rates of major complication (8%) combined with a short postoperative hospitalization (median 3 to 6 days) after SPRLC could result in lowering a surgeons’ threshold to restore intestinal continuity.

Our review and appraisal of the literature shows that type of single-port platform does not influence the favorable results after SPRLC. It appears that the postoperative results found in the literature are slightly better compared to the results found in our multicenter cohort. One reason might be that the majority of patients in this cohort have had open index surgery compared to the patients in the different cohorts in the literature (101/156 versus 61/131, *p* = 0.003). Another reason might be a possible publication or selection bias of those smaller case series in the literature. It seems reasonable and sensible to perform a certain patient selection (ASA 1–2 patients with low BMI and swift uncomplicated recovery after a laparoscopic procedure) when one is still adapting to a new technique [14]. Our cohort, on the other hand, contains mostly ASA 2–3 patients with a tendency towards being overweight. As time progressed and sufficient exposure was gained, SPRLC evolved from a novel technique, to the preferred approach which was applied to all left-sided colostomy reversal surgeries in ETH, CH and HRH. This might have resulted in an increase in conversions to multiport laparoscopy in the ETH cohort due to the inclusion of increasingly complex patients. Moreover, it needs to be stressed that the majority of these conversions consist of addition of one single 5 mm port, below the old laparotomy scar, not necessitating conversion to open laparotomy. Noteworthy is that CH cohort has an exceptionally high number of primary open approach in their mostly ASA 3 patients compared to others, which could be an explanation for their higher conversion rate during SPRLC.

Midline adhesiolysis or adhesiolysis in order to place trocars have become unnecessary when using the stoma fenestration as port, since the reversal of a left-sided colostomy takes place the left side of the abdomen alone. This advantage has been confirmed before [3, 13, 15, 23, 24]. Another advantage of SP is that it obviates the need to treat a concomitant complex abdominal wall defect or incisional hernia, this entire area can be left alone when using the stoma fenestration as access [15]. The use of a single-port with availability of multiple instruments through the stoma fenestration can be beneficial compared to conventional multiport laparoscopy, especially in patients after open surgery with extensive intra-abdominal adhesions. If needed, direct adhesiolysis can be safely performed first with those instruments through the single-port to ensure safe additional trocar placement elsewhere. This adhesiolysis to clear space for additional trocar placement is not always easy or feasible when using the conventional multiport laparoscopy.

SP surgery also has its down sides, it takes some adjustment from surgeons and surgical team to adapt to the off-centered vision and limited space to ‘triangulate’ the laparoscopic instruments. Despite this, we have shown before that experienced residents are able to perform this procedure under supervision, especially in centers with adequate experience and exposure in minimally invasive laparoscopic surgery [15].

This study is limited by the retrospective observational character; no randomization or case-matched comparisons have been carried out in this cohort. Another limitation of a retrospective observational study is the inability to include variables if they are not a part of the standard electronic patient records or operation reports, such as blood loss. Other factors such as enhanced recovery after surgery programs, low opioid anesthesia and analgesia or increasingly subspecialization of colorectal surgery with increasing laparoscopic index surgery could all have a part in the favorable results of SPRLC. Especially patient selection at surgeons’ preference (such as in the ZMC cohort) might result in exceptionally favorable results. It would be an interesting avenue of further research to evaluate if a standardized way of using the a single-port in the stoma fenestration with adding one or two additional ports from the start of the procedure would result in better peri- and post-operative results with possibly increased uptake of this technique.

This study confirms the safety, feasibility and favorable results of the use of single-port approach in the reversal of left-sided colostomy in different centers in Europe with laparoscopic experienced colorectal surgeons. The available literature on this topic support and show equally favorable results using single-port laparoscopy for left-sided colostomy reversal surgery.

## Supplementary Information

Below is the link to the electronic supplementary material.

Supplementary file1 (DOCX 15 kb)

Supplementary file2 (DOCX 12 kb)
