# Supplementary material for: Reversal of left-sided colostomy utilizing single-port laparoscopy a multicenter European audit and overview of the literature
Source: Surg Endosc. 2021 Jul 26;36(5):3389–97. doi: 10.1007/s00464-021-08657-x (PMC9001236; doi:10.1007/s00464-021-08657-x)
Supplement: Supplementary file 1 — Supplementary file1 (DOCX 15 kb) [file 464_2021_8657_MOESM1_ESM.docx]

Appendix 1 – overview of conversions and accompanying intra-operative complications or difficulties in SPRLC procedures in Elisabeth-TweeSteden Hospital

| Conversion | Multiport or Open | Intra-operative complication or difficulties |
| --- | --- | --- |
| Yes | Multiport | Ischemia of colon |
| Yes | Open | not enough length for anastomosis |
| Yes | Multiport | flexure mobilisation |
| Yes | Open | extensive adhesiolysis, positive air leak test |
| Yes | Open | rectal stump stapeler induced injury |
| Yes | Open | extensive adhesiolysis, serosal injury |
| Yes | Multiport | not enough length for anastomosis formation |
| Yes | Open | small bowel injury during port placement |
| Yes | Multiport | flexure mobilisation, adhesiolysis |
| Yes | Multiport | extensive adhesiolysis |
| Yes | Open | extensive adhesiolysis, inadvertant enterotomy |
| Yes | Multiport | positive air leak test |
| Yes | Open | positive air leak test |
| Yes | Open | extensive adhesiolysis, serosal injury |
| Yes | Multiport | positive air leak test |
| Yes | Open | frozen pelvis |
| Yes | Multiport | extensive adhesiolysis |
| Yes | Open | positive air leak test |
| Yes | Multiport | extensive adhesiolysis, positive air leak test |
| Yes | Multiport | extensive adhesiolysis |
| Yes | Open | positive air leak test |
| Yes | Open | extensive adhesiolysis |
| Yes | Multiport | extensive adhesiolysis, serosal injury |
| Yes | Open | extensive adhesiolysis, inadvertant enterotomy |
| Yes | Multiport | positive air leak test |
| Yes | Open | frozen pelvis |
| Yes | Multiport | extensive adhesiolysis, rectal stump stapeler induced injury |
