# Supplementary material for: Reversal of left-sided colostomy utilizing single-port laparoscopy a multicenter European audit and overview of the literature
Source: Surg Endosc. 2021 Jul 26;36(5):3389–97. doi: 10.1007/s00464-021-08657-x (PMC9001236; doi:10.1007/s00464-021-08657-x)
Supplement: Supplementary file 2 — Supplementary file2 (DOCX 12 kb) [file 464_2021_8657_MOESM2_ESM.docx]

Appendix 2 - Advanced search details

Search performed on pubmed.ncbi.nlm.nih.gov on 29-06-2020 for the last time.

((((((single-port) OR (single-incision)) OR (laparoscopy)) OR (trephine)) OR (stoma-site)) AND (((reversal) OR (colostomy take down)) OR (restoration))) AND (((hartmann) OR (hartmann procedure)) OR (hartmann's procedure)),,,"(((((""single-port""[All Fields] OR ""single-incision""[All Fields]) OR (((""laparoscopie""[All Fields] OR ""laparoscopy""[MeSH Terms]) OR ""laparoscopy""[All Fields]) OR ""laparoscopies""[All Fields])) OR (((((((""trephinated""[All Fields] OR ""trephinations""[All Fields]) OR ""trephine""[All Fields]) OR ""trephined""[All Fields]) OR ""trephines""[All Fields]) OR ""trephining""[MeSH Terms]) OR ""trephining""[All Fields]) OR ""trephination""[All Fields])) OR ((((""surgical stomas""[MeSH Terms] OR (""surgical""[All Fields] AND ""stomas""[All Fields])) OR ""surgical stomas""[All Fields]) OR (""stoma""[All Fields] AND ""site""[All Fields])) OR ""stoma site""[All Fields])) AND (((((((((((((""reversal""[All Fields] OR ""reversals""[All Fields]) OR ""reverse""[All Fields]) OR ""reversed""[All Fields]) OR ""reversely""[All Fields]) OR ""reverses""[All Fields]) OR ""reversibilities""[All Fields]) OR ""reversibility""[All Fields]) OR ""reversible""[All Fields]) OR ""reversing""[All Fields]) OR ""reversion""[All Fields]) OR ""reversions""[All Fields]) OR (((""colostomy""[MeSH Terms] OR ""colostomy""[All Fields]) OR ""colostomies""[All Fields]) AND ""take""[All Fields] AND ""down""[All Fields])) OR (((((((((((""restorability""[All Fields] OR ""restorable""[All Fields]) OR ""restorated""[All Fields]) OR ""restoration""[All Fields]) OR ""restoration s""[All Fields]) OR ""restorations""[All Fields]) OR ""restorative""[All Fields]) OR ""restoratives""[All Fields]) OR ""restore""[All Fields]) OR ""restored""[All Fields]) OR ""restores""[All Fields]) OR ""restoring""[All Fields]))) AND ((((""hartmann""[All Fields] OR ""hartmann s""[All Fields]) OR ""hartmanns""[All Fields]) OR (((""hartmann""[All Fields] OR ""hartmann s""[All Fields]) OR ""hartmanns""[All Fields]) AND (((((((""methods""[MeSH Terms] OR ""methods""[All Fields]) OR ""procedure""[All Fields]) OR ""methods""[MeSH Subheading]) OR ""procedures""[All Fields]) OR ""procedural""[All Fields]) OR ""procedurally""[All Fields]) OR ""procedure s""[All Fields]))) OR (((""hartmann""[All Fields] OR ""hartmann s""[All Fields]) OR ""hartmanns""[All Fields]) AND (((((((""methods""[MeSH Terms] OR ""methods""[All Fields]) OR ""procedure""[All Fields]) OR ""methods""[MeSH Subheading]) OR ""procedures""[All Fields]) OR ""procedural""[All Fields]) OR ""procedurally""[All Fields]) OR ""procedure s""[All Fields])))"

Results: 163

Filters: from 2011-2020

Results: 96
